# Ambulance patients with altered level of consciousness: a registry-based cohort study from the North Denmark Region in 2017–2021

**DOI:** 10.1186/s13049-026-01562-z

**Published:** 2026-01-23

**Authors:** Amalie Rabjerg Rosenkvist, Christine Norup Engstrøm, Ida Bromell Kindberg, Terese Miang Engstrup, Erika Frischknecht Christensen, Henrik Bøggild, Tim Alex Lindskou

**Affiliations:** 1 Centre for Prehospital and Emergency Research at Danish Centre for Health Services Research, Aalborg University Hospital, Aalborg, Denmark; 2 Department of Clinical Medicine, Aalborg University, Aalborg, Denmark; 3 Department of Health Science and Technology, Public Health and Epidemiology, Faculty of Medicine, Aalborg University, Aalborg, Denmark; 4 Research Data and Biostatistics, Aalborg University Hospital, Aalborg, Denmark

**Keywords:** Altered level of consciousness, Emergency Medical Services, Glasgow Coma Scale, Mortality

## Abstract

**Background:**

An altered level of consciousness (ALOC) in emergency patients is linked to poor outcomes. Glasgow Coma Scale (GCS), initially made for head injury patients, but often used in both trauma and other patients. GCS assesses consciousness, with scores ranging from 15 (fully aware) to 3 (deep coma). Assessing GCS is quickly done and does not require technical equipment, unlike other vital signs. This study aims to characterize unselected prehospital ALOC patients brought to a hospital and assess their mortality.

**Design:**

Retrospective observational cohort study.

**Methods:**

Patients transported by ambulance to a hospital in the North Denmark Region between 2017 and 2021, with a first registered GCS of 3–14, were included. The reason for calling the emergency number, subsequent ICD-10 diagnoses given at hospital, and raw and adjusted mortality, were reported.

**Results:**

Fifteen thousand two hundred thirty-five patients were included. One in eight ambulance patients had ALOC, most with a GCS score of 14. The primary reason for calling the emergency number was *decreased consciousness or paralysis,* except for those with a GCS score of 3–8, where the most common reason was *unconsciousness/possible cardiac arrest*. Most patients received non-specific diagnoses at hospital, which covers a wide range of symptoms and abnormal findings. Those with a GCS score of 3, most frequently were diagnosed with circulatory diseases, especially cardiac arrest and more diverse diagnostic picture among the others, including trauma, strokes, syncope, seizures and poisoning as the most frequent. Among patients with a GCS score of 3, 60% had died within 30 days, whereas 10% had died among patients with a GCS score of 14. The odds of mortality increased as GCS scores decreased, with a decrease from 14 to 9–13 more than doubled the odds of dying (OR 2.3, *p* < 0.001).

**Conclusion:**

Among unselected prehospital emergency patients brought to a hospital, every eight had ALOC. The patient group was heterogenic concerning the reasons for calling the emergency number and diagnoses given at hospital. ALOC was associated with high 30 days mortality, including 10% for GCS of 14. ALOC, also in milder degree, is a serious sign in a broad prehospital patient population.

**Supplementary Information:**

The online version contains supplementary material available at 10.1186/s13049-026-01562-z.

## Background

Level of consciousness is an essential indicator of patient health, with an altered level of consciousness (ALOC) associated with poor outcomes [1, 2]. As such, it is an important part of the early warning scores used for evaluating deteriorating patients [3, 4]. A reduced ALOC is important for identifying and prioritizing acute patients [2, 5].

The Glasgow Coma Scale (GCS) is often used to assess consciousness. A GCS score of 15 indicates a fully aware patient, while a score below 15 indicates reduced consciousness. An unconscious patient in a deep coma or a lifeless has a GCS score of 3 [6]. GCS was originally developed for use in patients with traumatic brain injury but is now often used for assessment of consciousness in other trauma patients as well as in non-trauma emergencies [6].

Most studies on ALOC patients are from Emergency Department (ED) settings, with the most common causes for ALOC being metabolic, cerebrovascular and infectious conditions [5, 7]. However, the patients may have been evaluated by the ambulance personnel prior to arriving at the hospital, and thereby already have received treatment during the prehospital phase. Few prehospital studies with various patient groups examine ALOC. Among non-trauma and trauma prehospital patients, US studies found that 12–27% had a low GCS score when the ambulance arrived on scene, defined as GCS below 12 or 15 [8, 9]. However, the entire unselected prehospital group of patients with ALOC is a complex group due to different etiologies causing ALOC [10].

Our aim is to characterize patients with ALOC at the arrival of the ambulance and estimate their mortality based on the level of ALOC.

## Methods

### Study design and setting

This is a retrospective observational cohort study, including data extracted from prehospital and in-hospital medical records in the North Denmark Region in the period 1 January 2017 to 31 December 2021. The study observed the cohort of patients brought to a hospital by ambulance, who had an altered level of consciousness.

The prehospital- and hospital system is managed by the five administrative regions of Denmark. The North Denmark Region has approximately 590,000 inhabitants and covers both urban and rural areas [11]. Healthcare in Denmark, including prehospital healthcare, is free of charge, as it is paid through taxes [12]. Every Danish citizen has a unique civil registration number, which makes it possible to link registries for the specific patient.

In Denmark, emergency calls involving a medical issue are forwarded to the Emergency Medical Coordination Centers, where healthcare professionals answer the call [12]. They assess the situation and the urgency with the aid of a criteria-based dispatch decision support tool, the Danish Index for Emergency Care. This tool divides medical events into 37 main criteria, corresponding to symptoms and situations [13]. As such, the Danish Index for Emergency Care reflects the primary reason for calling the emergency number. The healthcare professionals may dispatch an ambulance or similar units, with the option to bring the patient to an ED, a specialized trauma center or to treat and discharge them at the scene [12, 14].

Danish ambulances are manned by two persons, either a paramedic (3 year and 7-month education), or a paramedic with special competencies (currently three years experience as paramedic, in addition to a period of 20 weeks education). Both have authorization as healthcare professionals. Anesthesiologists are available as prehospital physicians in rapid response vehicles [12, 15].

All prehospital units use the electronic Prehospital Medical Record, for documentation. This contains patient information, main reason for calling the emergency number, vital signs, treatment, observations etc. Ambulance personnel perform primary and secondary assessments of the patients according to the ABCD principles, but do not systematically register the reason for encounter [16].

Whenever a patient has a hospital contact or is admitted, a diagnosis is assigned according to the International Classification of Diseases 10th Revision (ICD-10). This classification system is built upon main organ system or main pathology as main chapters of diseases, with detailed diagnoses as subchapters [17]. Most main chapters are classified as diseases in specific organ systems, whereas some are non-specific, covering symptoms and abnormal findings not elsewhere classified [17].

### Participants

Patients to whom an ambulance was dispatched following an emergency number call, and who subsequently were brought to a hospital. In addition, the patients were also required to have at least one registered GCS score. We included patients from the North Denmark Region in the period 1 January 2017 to 31 December 2021. All hospital contacts were included, both short visits (e.g., assessment and treatment in the ED) and cases where the patient was subsequently admitted.

Patients without a registered civil registration number were excluded to ensure follow-up. Patients below 15 years of age were also excluded, as GCS is different when assessing children [18]. As this study aims to investigate patients with ALOC, patients with a first measured GCS of 15 were considered conscious and thus excluded.

Patients with multiple contacts during the study period were included as separate occurrences. When assessing mortality, only the last prehospital contact during the five-year study period was included for patients with multiple contacts.

### Data sources and data management

Information on the main reason for calling the emergency number and the first measured GCS on-scene, were obtained from the Prehospital Medical Record. Age, sex and date of death were retrieved from the Danish Civil Registration System. Diagnoses given at the hospital were obtained from the regional Patient Administrative System.

### Statistical analysis

Patients with ALOC at the arrival of the ambulance were stratified into four groups depending on their first measured GCS score (GCS 3, 4–8, 9–13, and 14) [3]. All analyses were stratified according to these four groups.

Median and interquartile range were used to describe the distribution of age. Sex, GCS scores, main reason for calling the emergency number, and ICD-10 diagnostic chapters were described by absolute numbers and proportions. Kruskal–Wallis and Chi-Square tests were used to compare the distribution of age and. Mortality was defined as the proportion of patients who were dead after 48 h, 7 days, and 30 days from the emergency call. Survival was described using Kaplan–Meier curves. Logistic regression models were used to estimate the odds ratios for mortality with 95% confidence intervals (95% CI), adjusting for age, sex and main reason for calling the emergency number. Patients with a GCS of 14 were used as the reference group. The statistical level was set at α = 0.05.

Statistical analyses were performed using StataMP 17.0 (Stata Corporation, College Station, Texas, USA).

## Results

**Fig. 1:**
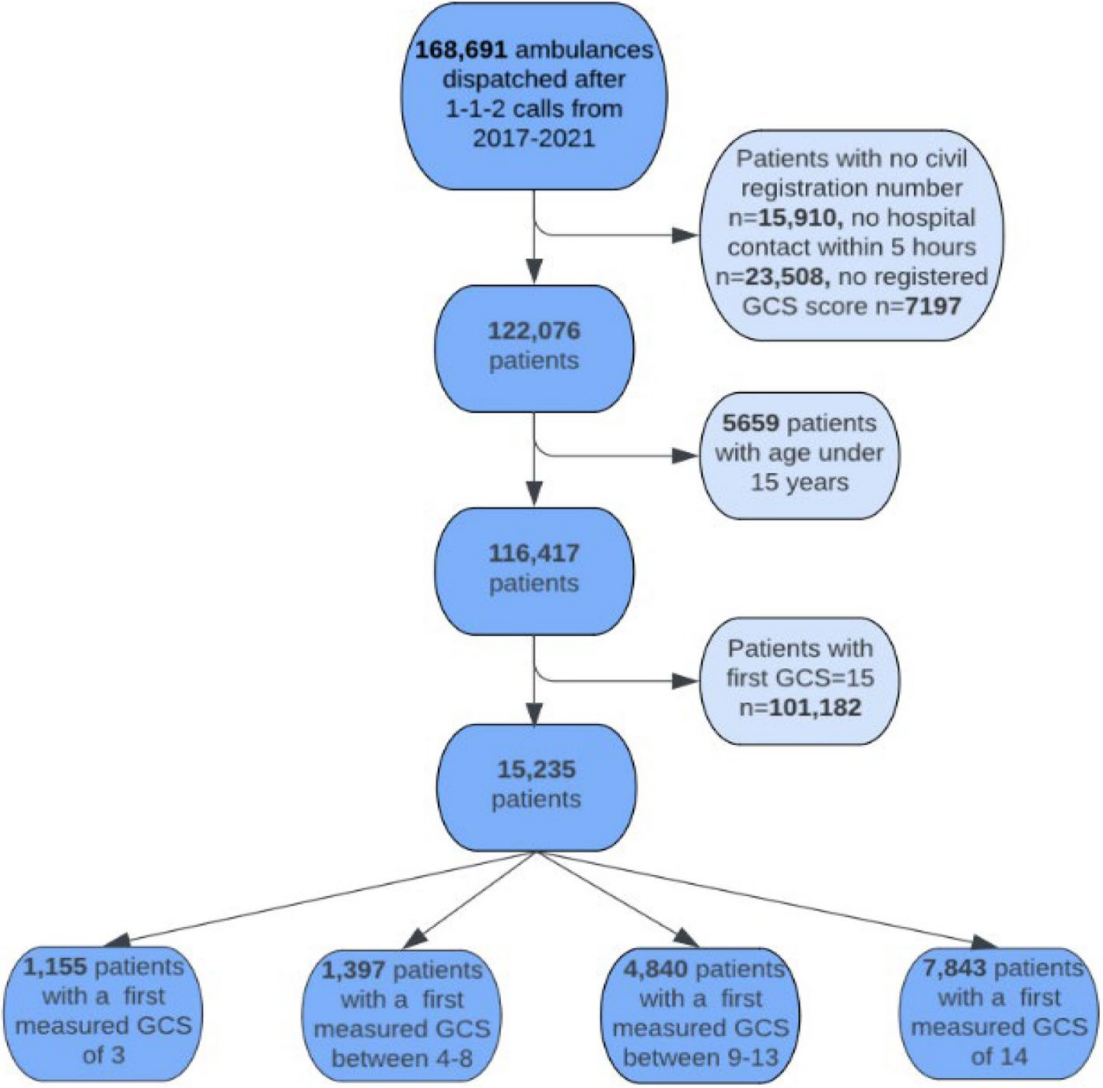
Ambulance patients with ALOC in the years 2017–2021 in the North Denmark Region. Flowchart showing the exclusion of patients. Patients were stratified into four groups by first measured GCS

From 2017–2021, 168,691 patients had an ambulance dispatched following an emergency call. After excluding patients who were not transported to a hospital, had no civil registration number or registered GCS score, were under the age of 15, or had normal GCS, we included 15,235 ALOC patients for analyses (Fig. 1).

Half of the patients had an initial GCS of 14, approximately a third in the range of 9–13 and 10% between 4–8 (Table 1). Eight percent had an initial GCS of 3. The median age was 69 years (Q1-Q3: 49–81). All ALOC groups had similar age distributions. Females constituted 44% of the patients with ALOC. Statistically significantly more males had a GCS of 3 compared to females (*p* < 0.01) (Table 1).

**Table 1:** Characteristics of 15,235 ambulance patients with altered level of consciousness from 2017—2021 stratified by first measured Glasgow Coma Scale score including information on whether vital signs were recorded in the ambulance. Information is number (percentage), except for age, which is quartiles

	**GCS 3** (*n* = 1,155)	**GCS 4–8** (*n* = 1,397)	**GCS 9–13** (*n* = 4,840)	**GCS 14** (*n* = 7,843)	**All ALOC patients** (*n* = 15,235)
Sex, women	442 (38.3%)	639 (45.7%)	2,143 (44.3%)	3,478 (44.4%)	6,702 (44.0%)
Age, median (Q1-Q3)	67 (52–77)	67 (47–80)	69 (49–81)	69 (48–81)	69 (49–81)
**Main reason for calling the emergency number**					
Decreased consciousness—paralysis	50 (4.3%)	216 (15.5%)	1,190 (24.6%)	1,983 (25.3%)	3,439 (22.6%)
Unconscious adult/possible cardiac arrest	545 (47.2%)	338 (24.2%)	578 (11.9%)	370 (4.7%)	1,831 (12.0%)
Unclarified problem	72 (6.2%)	147 (10.5%)	585 (12.1%)	979 (12.5%)	1,783 (11.7%)
Seizures	114 (9.9%)	236 (16.9%)	500 (10.3%)	739 (9.4%)	1,589 (10.4%)
Accidents	16 (1.4%)	47 (3.4%)	267 (5.5%)	936 (11.9%)	1,266 (8.3%)
Alcohol—poisoning—overdose	61 (5.3%)	129 (9.2%)	474 (9.8%)	501 (6.4%)	1,165 (7.6%)
Breathing difficulties	71 (6.1%)	85 (6.1%)	381 (7.9%)	446 (5.7%)	983 (6.5%)
Remaining reasons	167 (14.5%)	129 (9.2%)	666 (13.8%)	1,564 (19.9%)	2,526 (16.6%)
None^a^	59 (5.1%)	70 (5.0%)	199 (4.1%)	325 (4.1%)	653 (4.3%)
**ICD-10 Diagnosis**					
Non-specific diagnoses^b^	251 (21.7%)	333 (28.8%)	1,276 (26.4%)	2,295 (29.3%)	4,155 (27.3%)
Injuries and poisoning	131 (11.3%)	186 (13.3%)	697 (14.4%)	1,729 (22.1%)	2,743 (18.0%)
Circulatory	483 (41.8%)	221 (15.8%)	743 (15.4%)	776 (9.9%)	2,223 (14.6%)
Nervous system	92 (8.0%)	185 (13.2%)	440 (9.1%)	631 (8.1%)	1,348 (8.8%)
Mental disorders	51 (4.4%)	148 (10.6%)	470 (9.7%)	597 (7.6%)	1,266 (8.3%)
Respiratory	75 (6.5%)	110 (7.9%)	368 (7.6%)	489 (6.2%)	1042 (6.8%)
Metabolic	30 (2.6%)	94 (6.7%)	303 (6.3%)	336 (4.3%)	763 (5.0%)
Infections	14 (1.2%)	58 (4.2%)	226 (4.7%)	372 (4.7%)	670 (4.4%)
Genitourinary	8 (0.7%)	24 (1.7%)	137 (2.8%)	260 (3.3%)	429 (2.8%)
Digestive system	11 (1.0%)	23 (1.7%)	89 (1.8%)	189 (2.4%)	312 (2.0%)
Remaining diagnosis chapters	9 (0.8%)	15 (1.1%)	91 (1.9%)	169 (2.2%)	284 (1.9%)
**Total patients**	**1,155 (7.6%)**	**1,397 (9.2%)**	**4,840 (31.8%)**	**7,843 (51.5%)**	**15,235 (100%)**

^a^Patients with no registered Danish Index category

^b^Combined ICD-10 main chapter XVIII “symptoms and signs” and main chapter XXI “other factors”

For all ALOC patients, *Decreased consciousness – paralysis* was the most frequent reason for calling the emergency number, followed by *Unconscious adult/possible cardiac arrest*, and *Unclarified problem*. These three constituted nearly half (46.3%) of all reasons for calling the emergency number (Table 1).

Among patients with GCS 9–13 and GCS 14, the most frequent reason for calling the emergency number was *Decreased consciousness—paralysis,* whereas *Unconscious adult/possible cardiac arrest* was the most frequent in the GCS 4–8 and GCS 3 groups. *Accidents* were more common among the GCS 14 patients, while *Seizures* were more common among patients with GCS 4–8. Patients with GCS 3 were less frequently categorized as *Unclarified problem* compared to the other groups (6.2% vs 20.5–12,5%) (Table 1).

For all ALOC patients, the most common diagnoses given at hospital were *non-specific diagnosis* (ICD-10 main chapter XVIII *symptoms and signs* and main chapter XXI *other factors*), followed by *injuries and poisoning* and *circulatory diseases*, for a combined 59.9% of all diagnoses. *Non-specific diagnoses* were most frequently associated with observation due to suspected disease*,* seizures, syncope or lipothymia due to different reasons (Table 1 and supplementary).

For patients with GCS 3, the main chapter *circulatory diseases* was most common, where the specific sub-diagnoses were mainly related to cardiac arrest (54.1% including cardiac arrest with successful resuscitation) For GCS 4–8, GCS 9–13, *circulatory diseases* were also prominent, albeit the specific sub-diagnoses were more frequently related to stroke (46.1%—58.8%) (Table 1 and supplementary).

Patients with GCS 14 more frequently received diagnoses from the main chapter *injuries and poisoning*, with the specific sub-diagnoses relating to concussions and lesions (Table 1 and supplementary).

**Fig. 2:**
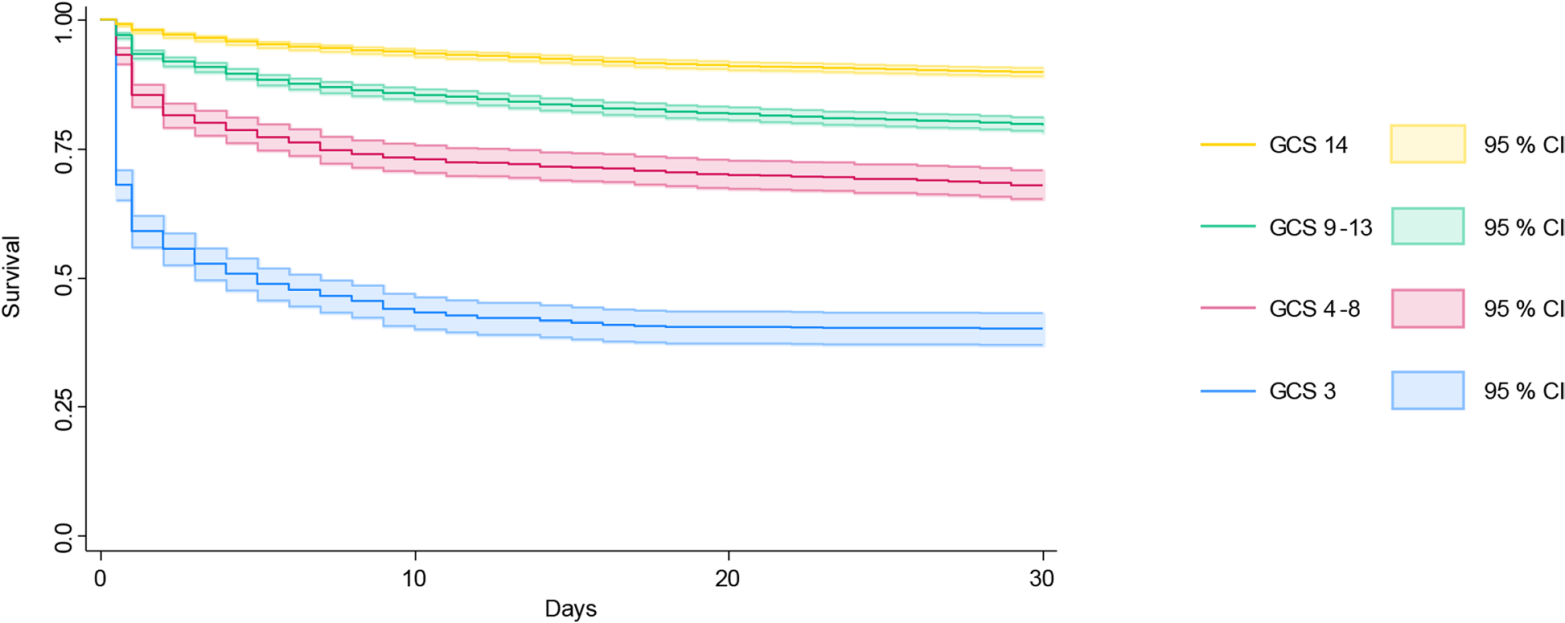
Survival described by Kaplan–Meier curves and confidence intervals for up to 30 days, stratified by Glasgow Coma Scale score at the first prehospital measurement

During the study period 969 patients died within 48 h of the emergency call, corresponding to a mortality of 7.8%. Within 7 days, 13.6% patients had died and 19.4% died within 30-days (Fig. 2, Table 2). The highest mortality was seen among patients with a GCS of 3 (Fig. 2, Table 2).

**Table 2:** Mortality after 48 h, 7 days, and 30 days. Stratified by the initial Glasgow Coma Scale

	**GCS 3**	**GCS 4–8**	**GCS 9–13**	**GCS 14**	**All ALOC patients**
48-h mortality (total % of group)	421 (41.1%)	158 (14.6%)	255 (6.7%)	135 (2.1%)	969 (7.8%)
7-day mortality (total % of group)	550 (53.7%)	273 (25.2%)	500 (13.1%)	357 (5.5%)	1680 (13.6%)
30-day mortality (total % of group)	614 (59.8%)	346 (32.0%)	782 (20.6%)	661 (10.2%)	2403 (19.4%)

Compared to patients with GCS 14, mortality increased as GCS decreased (Table 3). The same pattern was seen when adjusting for age, sex and main reason for calling the emergency number, which all had a significant effect on mortality. The adjusted odds ratio for mortality was considerably higher for the group with GCS 3 at both 48-h, 7 days, and 30 days, compared to the unadjusted odds ratios.

**Table 3:** Mortality according to altered level of consciousness compared to patients with initial Glasgow Coma Scale (GCS) of 14, unadjusted, adjusted for age and sex, and further for Danish Index for Emergency Care criteria. Odds ratio (OR) and 95% confidence intervals (95% CI) estimated from logistic regression

	**48 h mortality**			**7 days mortality**			**30 days mortality**		
	**OR**	**95% CI**	***P*-value**	**OR**	**95% CI**	***P*-value**	**OR**	**95% CI**	***P*-value**
Unadjusted OR									
GCS 3	32.6	26.4—40.3	< 0.001	19.8	16.8—23.3	< 0.001	13.1	11.3—15.2	< 0.001
GCS 4–8	8.0	6.3—10.2	< 0.001	5.8	4.9—6.9	< 0.001	4.1	3.6—4.8	< 0.001
GCS 9–13	3.4	2.7—4.2	< 0.001	2.6	2.3—3.0	< 0.001	2.3	2.0—2.5	< 0.001
GCS 14	1			1			1		
OR adjusted by age and sex									
GCS 3	40.9	32.8—51.0	< 0.001	28.6	23.9—34.3	< 0.001	20.7	17.4—24.5	< 0.001
GCS 4–8	8.6	6.8—11.0	< 0.001	6.7	5.5—8.0	< 0.001	5.0	4.2—5.9	< 0.001
GCS 9–13	3.4	2.7—4.2	< 0.001	2.6	2.3—3.0	< 0.001	2.4	2.1—2.7	< 0.001
GCS 14	1			1			1		
OR adjusted by age, sex and Danish Index									
GCS 3	35.1	27.7—44.5	< 0.001	24.9	20.6—30.2	< 0.001	18.2	15.2—21.8	< 0.001
GCS 4–8	8.2	6.3—10.5	< 0.001	6.5	5.4—7.9	< 0.001	4.9	4.1—5.8	< 0.001
GCS 9–13	3.3	2.7—4.1	< 0.001	2.6	2.2—3.0	< 0.001	2.3	2.1—2.6	< 0.001
GCS 14	1			1			1		

## Discussion

### Principle findings

This study found that every eighth of ambulance patients with a hospital contact had ALOC at ambulance arrival, with nearly half having a GCS score of GCS 14.

The most frequent reason for calling the emergency number was *Decreased consciousness—paralysis*, except for patients with a lower GCS score, where the most common reason for the call was *Unconscious adult/possible cardiac arrest*. These two constituted 43.6% of the entire group, with seizures and alcohol-poisoning-overdose accounting for another 18%, meaning that in about two thirds of the cases, the ALOC was perceived as such when assessing the emergency call.

The diagnostic picture was diverse, and regardless of the first measured GCS, most patients received an in-hospital diagnosis within the *non-specific diagnosis* main chapters The exception was patients with GCS 3 where the main chapter *circulatory diseases* were most frequent*,* the majority being cardiac arrest or cardiac death. But even within this group of patients with deep ALOC seizures and poisonings were found.

The most diverse diagnostic patten was seen among patients with GCS 14, with concussions, seizures and syncope as the most frequent, but also with very serious diseases such as stroke. For patients with GCS between 4 and 13 a similar pattern was seen with increasingly serious conditions concerning mortality. This was high, even among patients with GCS 14 the 30- day mortality was 10%, and 60% among patients with GCS 3. As GCS score decreased, the odds of mortality at both 48 h, 7 days, and 30 days increased.

### Comparison with other prehospital studies

An Australian study included 129,192 emergency ambulance patients whose level of consciousness was assessed and registered (Alert, Confused, Drowsy, Voice Response, Pain Response, Unresponsive) [2]. Excluding alert patients, the overall most prominent used Medical Priority Dispatch System protocol was *Unconscious/Fainting (near), Stroke (cerebrovascular accident), Overdose/Poisoning (ingestion), Breathing problems,* and *Convulsions/Fitting* [2]. A similar overall distribution was present in the current study. Likewise, among unresponsive patients the most prominent Medical Priority Dispatch System protocol was, *Cardiac or respiratory arrest/death*, corresponding to the main reason for calling 1–1–2 *Unconscious adult/possible cardiac arrest*among patients with a GCS of 3 in the current study [2]. Belcher et al. found that level of consciousness was an overall good predictor of acuity, similarly to our study [2].

A small study by Björkman et al. found that the most common etiologies of prehospital ALOC (GCS < 12) in 306 non-trauma patients were seizures, diabetes, intoxication and no obvious cause which among others included cerebrovascular diagnoses and infection [1].

Our study found that patients in all GCS groups were often diagnosed within the ICD-10 main chapters covering *non-specific diagnoses*. Studies from the North Denmark Region evaluated the diagnostic patterns in the entire group of prehospital patients [19, 20]. Similar to our results, these studies found the use of non-specific diagnoses prominent and with a tendency towards increased use over time [19, 20]. which is remarkable that patients with serious symptoms, like ALOC, do not receive a more specific diagnosis. This might be influenced by the patient regaining some level of consciousness following the prehospital phase at hospital. The underlying condition of the symptoms could be unknown and thus registered as ‘*Observation due to suspected disease or condition*’, the dominant non-specific diagnosis across all GCS subgroups. Among others, observation for concussion is frequently registered as this.

Also, Warwick et al. found that a GCS score below 14 was the best prehospital predictor of both ED and in-hospital mortality [9]. Furthermore, they investigated OR of ED deaths and in-hospital deaths among patients with a GCS score below 14, and found a OR of 10.8 and 4.4 respectivly [9]. As such, a GCS score below 14 was related to higher odds of dying, and the lower the GCS score, the higher the odds of mortality, especially in the beginning of their admission [9]. This aligns with both the study by Belcher et. al and the current study [2].

### Strengths and limitations

One of the strengths of this study was the linked access to health data by using the unique civil registration number. This makes it possible to combine data from the prehospital setting with the diagnoses given at the hospital, enabling full follow-up, including death within 30 days. The study design furthermore enabled examination of data from a large patient population, resulting in precise estimates. The unselected prehospital patient group, covering both trauma and non-trauma patients, strengthen the external validity. Another strength was that access to the healthcare system is free in Denmark, which minimizes selection bias, as all patients have equal access to healthcare services and would be included if calling an ambulance.

The results of the present study are considered generalizable to Scandinavia, due to the similarities in the emergency medical service systems with dispatch protocols, quality assurance and publicly funded healthcare. Local variations are present, but the effects are considered minimal, supporting the interpretation of the results in a broader Scandinavian context [15].

A limitation of this study was the prevalence of missing data for the civil registration number. Missing registration could potentially be due to the unconsciousness of the patient. Thereby, there might be an association between having missing data and ALOC, but the risk seems small, as the registration number would normally by identified at the hospital, and thus updated. As such, the number of ALOC patients may be underestimated, but the effect is expected to be limited. Furthermore, tourists do not have a civil registration number and would thereby not be included in the study population.

GCS is among the vital parameters with highest completion in the prehospital medical record, which is one of the strengths of focusing on GCS [16]. In our setting there was 5% missing registration of GCS measurement, possibly due to the hectic environment in case of a critical case in the ambulance, or in case of minor severity not deemed necessary to assess GCS scores. This could be the case for patients with and without ALOC. As both patients with severe ALOC and mild ALOC might have been excluded, this would probably lead to minimal bias.

The assessment of GCS is done by educated and trained healthcare professionals. Furthermore, the presenting symptom of the patient is not systematically registered by ambulance personnel. As such, the main reason for calling assessed at the dispatch center (DI criteria) is the only available source of the patients’ presenting symptoms.

Not all patients who call for an ambulance are hospitalized, as some conditions are easily treatable according to EMS protocols, or in no need of treatment, leading patients to be discharged at the scene. Examples include hypoglycemic patients, who after receiving glucose injections discharged at the scene, and patients with known epilepsy after anti-seizure treatment. We do not consider this as influencing our main results, except for a probably small underestimation of these diagnostic groups in our study.

Furthermore, we only included ambulance patients from North Denmark hospitals, and some patients with severe symptoms might have been transported to more specialized hospitals or by helicopter. This would possibly result in patients with a low GCS not being included in this study. However, this proportion is small as the region has a university hospital including trauma-center level 1 and advanced stroke interventions and therefore should not affect the results significantly.

## Conclusion

Our findings demonstrate that ALOC is a frequent and clinically significant condition in the prehospital setting, affecting 13% of ambulance patients transported to hospitals in the North Denmark Region. Even mild ALOC with a GCS of 14 was associated with a 10% 30-day mortality, while patients with GCS 3 had nearly 60% mortality, confirming GCS as a strong prognostic marker. The patient group was heterogeneous, with diagnoses ranging from cardiac arrest and stroke to seizures, poisoning, and trauma, underscoring the complexity of managing ALOC. Given the similarities in emergency medical systems across Scandinavia, these results are highly relevant for regional planning and quality improvement initiatives. The study adds epidemiological data and mortality benchmarks for prehospital ALOC, supporting its use as a key triage parameter and highlighting the need for systematic assessment and prioritization in Scandinavian EMS systems.

## Supplementary Information

Supplementary Material 1.

## Data Availability

The data that support the findings of this study are available from the authors, but restrictions apply to the availability of these data, which were used following and approval for handover of medical records from The North Denmark Region for the current study and so are not publicly available. Data are, however, available from the authors upon reasonable request and with permission from The North Denmark Region.

## Abbreviations

ALOC: Altered level of consciousness
GCS: Glasgow Coma Scale
ED: Emergency department
ICD-10: International Classification of Diseases 10th Revision
