# Supplementary material for: Ambulance patients with altered level of consciousness: a registry-based cohort study from the North Denmark Region in 2017–2021
Source: Scand J Trauma Resusc Emerg Med. 2026 Jan 23;34:40. doi: 10.1186/s13049-026-01562-z (PMC12910908; doi:10.1186/s13049-026-01562-z)
Supplement: Supplementary file 1 — Supplementary Material 1. [file 13049_2026_1562_MOESM1_ESM.docx]

| **GCS 3** | | |
| --- | --- | --- |
| **Circulatory** (n = 483) | **Non-specific diagnoses** (n = 251) | **Injuries and poisoning** (n=131) |
| Cardiac arrest, NOS* (169; 35.0%) | Observation due to suspected disease or condition, NOS* (97; 38.6%) | Drug poisoning, NOS* (10; 7.6%) |
| Cardiac arrest with successful resuscitation (92; 19.1%) | Cardiac death, NOS* (81; 32.3%) | Acute traumatic subdural hemorrhage (8; 6.1%) |
| Sudden cardiac death (28; 5.8%) | Respiratory arrest (11; 4.4%) | Benzodiazepine derivative poisoning (8; 6.1%) |
| Atrial fibrillation (24; 5.0%) | Seizures, NOS* (11; 4.4%) | Morphine poisoning (5; 3.8%) |
| Acute myocardial infarction, NOS* (23; 4.8%) | Contact with health services under other circumstances (7; 2.8%) | Opioid poisoning (5; 3.8%) |
|  |  |  |
| **GCS 4-8** | | |
| **Non-specific diagnoses** (n = 333) | **Circulatory** (n = 221) | **Injuries and poisoning** (n = 186) |
| Observation due to suspected disease or condition, NOS* (145; 43.5%) | Hemorrhagic stroke (50; 22.6%) | Poisoning, NOS* (17; 9.1%) |
| Seizures, NOS* (54; 16.2%) | Ischemic stroke (37; 16.7%) | Concussion (14; 7.5%) |
| Syncope or collapse (20; 6.0%) | Apoplexia cerebri, NOS* (15; 6.8%) | Acute traumatic subdural hemorrhage (14;7.5%) |
| Contact with health services under other circumstances (11; 3.3%) | Cardiac arrest with successful resuscitation (10; 4.5%) | Traumatic subdural hemorrhage (8; 4.3%) |
| Observation due to suspected diseas of the nervous system (9; 2.7%) | Cardiac arrest, NOS* (10; 4.5%) | Drug poisoning, NOS* (8; 4.3%) |
|  |  |  |
| **GCS 9-13** | | |
| **Non-specific diagnoses** (n = 1,276) | **Circulatory** (n = 743) | **Injuries and poisoning** (n = 697) |
| Observation due to suspected disease or condition, NOS* (534; 41.8%) | Ischemic stroke (297; 40.0%) | Concussion (73; 10.5%) |
| Seizures, NOS* (165; 12.9%) | Hemorrhagic stroke (99; 13.3%) | Alcohol poisoning, NOS* (39; 5.6%) |
| Observation due to suspected diseas of the nervous system (71; 5.6%) | Apoplexia cerebri, NOS* (41; 5.5%) | Benzodiazepine derivative poisoning (38; 5.5%) |
| Syncope or collapse (64; 5.0%) | Intracerebral hemorrhage in one cerebral hemisphere, NOS* (25; 3.4%) | Drug poisoning, NOS* (36; 5.2%) |
| Contact with health services under other circumstances (46; 3.6%) | Atrial fibrillation or atrial flutter, NOS* (16; 2.1%) | Poisoning, NOS* (29; 4.2%) |
|  |  |  |
| **GCS 14** | | |
| **Non-specific diagnoses** (n= 2,295) | **Injuries and poisoning** (n = 1,729) | **Circulatory** (n = 776) |
| Observation due to suspected disease or condition, NOS* (817; 35.6%) | Concussion (347; 20.1%) | Ischemic stroke (267; 34.4%) |
| Seizures, NOS* (250; 10.9%) | Open lesion of the scalp (109; 6.3%) | Hemorrhagic stroke (64; 8.2%) |
| Syncope or collapse (125; 5.4%) | Superficial lesion of the scalp (74; 4.3%) | Apoplexia cerebri, NOS* (42, 5.4%) |
| Observation due to suspected diseas of the nervous system (86; 3.7%) | Traumatic rhabdomyolysis (55; 3.2%) | Atrial fibrillation or atrial flutter, NOS* (24; 3.1%) |
| Confusion, NOS* (68; 3.0%) | Open lesion of the head, NOS* (41; 2.4%) | Subarachnoid hemorrhage (19, 2.4%) |

*Appendix 1: 3 most common ICD-10 chapters for each group stratified by GCS and 5 most common subdiagnoses for each chapter. Subdiagnoses given as (number; percentage).
*Not otherwise specified*
